# CREDO: Highly confident disease-relevant A-to-I RNA-editing discovery in breast cancer

**DOI:** 10.1038/s41598-019-41294-y

**Published:** 2019-03-25

**Authors:** Woochang Hwang, Stefano Calza, Marco Silvestri, Yudi Pawitan, Youngjo Lee

**Affiliations:** 1 Data Science for Knowledge Creation Research Center, Seoul National University, Seoul, South Korea; 2 Department of Statistics, Seoul National University, Seoul, South Korea; 3 Department of Medical Epidemiology and Biostatistics, Karolinska Insitutet, Stockholm, Sweden; 4 Department of Molecular and Translational Medicine, University of Brescia, Brescia, Italy; 5 Department of Applied Research and Technical Development, Fondazione IRCCS, Istituto Nazionale dei Tumori, Milan, Italy

## Abstract

Adenosine-to-Inosine (A-to-I) RNA editing is the most prevalent post-transcriptional modification of RNA molecules. Researchers have attempted to find reliable RNA editing using next generation sequencing (NGS) data. However, most of these attempts suffered from a high rate of false positives, and they did not consider the clinical relevance of the identified RNA editing, for example, in disease progression. We devised an effective RNA-editing discovery pipeline called CREDO, which includes novel statistical filtering modules based on integration of DNA- and RNA-seq data from matched tumor-normal tissues. CREDO was compared with three other RNA-editing discovery pipelines and found to give significantly fewer false positives. Application of CREDO to breast cancer data from the Cancer Genome Atlas (TCGA) project discovered highly confident RNA editing with clinical relevance to cancer progression in terms of patient survival. RNA-editing detection using DNA- and RNA-seq data from matched tumor-normal tissues should be more routinely performed as multiple omics data are becoming commonly available from each patient sample. We believe CREDO is an effective and reliable tool for this problem.

## Introduction

Post-transcriptional modifications are essential for normal gene expression and tissue development. RNA editing, i.e., base conversion, insertion, or deletion of RNA molecules, is one of the most prevalent of such modifications; it plays crucial roles in fine-tuning gene expression and provides the diversity of the transcriptome^1^. A-to-I RNA editing, i.e., conversion of adenosine (A) to inosine (I), is most prevalent events; inosine is interpreted as guanosine (G) by reverse transcription and the translational machinery, so A-to-I editing is also known as A-to-G editing. Studies have been performed to discover the pathological relationship between RNA editing and diseases. It was reported that aberrant ADAR, a family of RNA’s adenosine to inosine editing enzymes, regulation and aberrant RNA-editing profiles are associated with many diseases including neurological disorders and cancers^2–8^. It was also reported that changing a single RNA editing level can drive cancer progression^3^. Sagredo *et al*. showed that RNA editing in 3′ UTRs and exonic regions are increased in breast cancer cells compared to immortalized non-malignant cells, and high-level of ADAR1 expression was associated with worse clinical outcome and increased editing in 3′UTRs^9^. It is also previously shown that RNA editing and ADAR expression are significantly altered in most cancer types and increased editing activity is associated with patient survival^10^.

Thus RNA-editing loci could be promising therapeutic targets. However, most of these functional studies were limited to individual RNA-editing events. Identification of RNA editing at the global transcriptomic level should broaden our insights to the post-transcriptional modification landscape and its pathological relevance to disease development. Therefore, an effective RNA-editing discovery in diseases should comprise a highly confident RNA editing identification and an investigation of pathological or clinical relevance of the spotted candidate RNA-editing events.

Advances of next generation sequencing (NGS) allows discovery of RNA-editing events at global transcriptomic level and several RNA editing discovery methods have been developed^11–15^. Because of the high statistical noise in NGS data and the incidental and potentially mosaic RNA-editing event, i.e. occurring in a subset of cell in specific individuals, identification of RNA-editing loci at transcriptomic level is highly susceptible to numerous false positives^16–19^. Many studies have often focused on single samples, so it is unclear whether the identified editing events are individual-specific or prevalent in the general population. Furthermore, the majority of the identified editing loci were in noncoding and repetitive-element regions without obvious functional relevance.

Piskol *et al*. developed an approach that can identify genomic variants reliably from RNA-seq. They identified genomic variants from RNA-seq data proposing a modified mapping procedure that can avoid misalignment of split reads and a variant filtering process that can prune spurious variants called in error-prone regions, repeated region, splice sites, homopolymer runs, etc.^20^. However, Hsiao *et al*. showed that the vast majority of RNA editing occurs co-transcriptionally prior to polyadenylation in human cells. It proposes the hypothesis that many RNA editing events may alter splicing by changing sequences on the splicing sites or by ADAR’s interaction with dsRNA structure^21^. They claim that the RNA editing in splice regions plays a crucial role in RNA maturation and splicing process. Hard filtering of RNA editing in splice regions can lose this important information.

Recently, Han *et al*. did an impressive work on RNA editing using extensive cancer sample data from the TCGA project^22^. Some functional and clinical analyses were performed for the identified RNA-editing events among various cancer samples. A number of RNA-editing events showed significant functional and clinical relevance in some cancer sites. However, they only attempted to find informative RNA-editing events from previously reported editing sites (in RADAR)^14^ rather than to identify novel disease-specific RNA-editing events. In addition, they did not find any RNA-editing events relevant to disease progression in breast cancer.

We developed an effective pipeline called CREDO with novel statistical modules that capture highly confident RNA-editing events in the TCGA breast-cancer samples. When compared to three other RNA-editing identification pipelines CREDO produced significantly fewer false positives as measured by the occurrence in the dbSNP database. Furthermore, CREDO discovered highly confident RNA-editing loci that were recurrent in multiple samples and clinically associated with breast-cancer patient survival.

## Materials and Methods

### Data

A total of 60 breast cancer patients that have both Exome-seq and RNA-seq from normal and tumor matched samples were chosen in the TCGA breast cancer database^23^. Raw sequencing data in FASTQ/bam format and clinical data of these patients were obtained from the TCGA data portal. The sequencing data can be downloaded from TCGA Data Portal (https://portal.gdc.cancer.gov/).

### Sequencing Data Analysis

**Figure 1:**
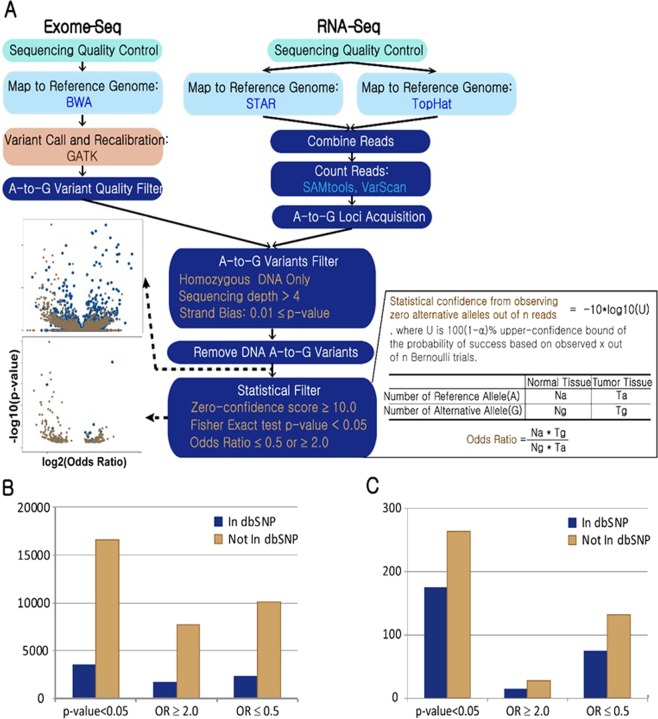
(**A**) CREDO RNA-Editing discovery pipeline. Novel statistical RNA-editing discovery modules are colored in dark blue. Scatter plots show A-to-G loci before and after the statistical discovery filter application. (**B**) Number of editing sites discovered by CREDO. Zero-confidence score ≥ 10.0 (C) Number of editing sites identified in more than 4 individuals out of the loci in (**B**).

CREDO RNA editing discovery pipeline is outlined in Fig. 1A. CREDO requires sequencing data of RNA and Exome of normal and disease matched sample for a case. Processes from ‘sequencing quality control’ to ‘Zero-confidence score’ computation in Fig. 1A should be carried out for each sample, i.e., normal or disease sample, independently for each individual. Then, confident RNA editing will be discovered by comparing between normal and tumor matched samples. Novel statistical RNA editing discovery modules are colored in dark blue. Base quality of reads in fastq files was checked and controlled (FastQC^24^, Galaxy^25^).

### Exome-seq Analysis

High quality reads were mapped to the reference genome (GRCh37) by BWA allowing 3 edit distance^26^. Duplicate reads were marked and removed to reduce bias from library preparation, e.g., PCR artifact (Picard)^27^. Insertion and deletion (indels) in an individual’s genome compared against the reference genome causes many mismatches around them and may mislead variants discovery. Therefore, the reads around indels were realigned locally (GATK IndelRealigner^28^). Variants are identified based on base quality scores. The scores produced by the machine are distorted by various sources of systematic technical errors. So, base quality scores are adjusted before variant calling using GATK BaseRecalibrator (the dbSNP build 138 and 1000-genome gold standard indels were used). DNA variants were identified and filtered by GATK HaplotypeCaller. Confident DNA variants were called by setting “stand_call_conf” to 30.0 and “stand_emit_conf” to 10.0. Obtained variants were recalibrated through GATK variant recalibration tool (VariantRecalibrator). It develops a covariance model between SNP call annotations (DP, QD, FS, SOR, MQ, etc.) and the probability that a SNP is a true genetic variant versus a sequencing or data processing artifact. The SNPs from Hapmap Project 3 and 1000 genome Omni 2.5 M SNP chips were used as true sites. It uses the covariance model built based on the true SNP sites and assign new variant quality scores. Only high-quality A-to-G homozygous DNA variants supported by more than 4 mapped reads and calling quality ≥50 were retained for further analyses.

### RNA-seq Analysis

Since different RNA-seq aligners may produce different mapping results^29^, we employed two of the best performing aligners (STAR^30^ and TopHat^31^) in order to overcome the shortcomings of each aligner. Maximum 3 mismatches (STAR) and 3-base edit distance (TopHat) were allowed in the mapping. After mapping, duplicate reads were marked and removed as in the previous section. For the following process, reads marked with ‘N’ symbol were removed, and overhanging regions into the intronic regions were hard-clipped to avoid the pitfalls caused by these artifacts (GATK Split ‘N’Trim). Base quality scores were recalibrated (GATK BaseRecalibrator). Mapped reads from both aligners were combined as follows. The read mapped only by an aligner on unique genomic location was retained. The best-scored read was retained when there are identical reads mapped on the same genomic location by both aligners. The highest-scored read out of the same reads mapped on different genomic loci by different aligners was retained. After combining information from the two aligners, the number of reads mapped on each genomic location was computed (SAMtools^32^, VarScan^33^). Only the loci that have adenosine (A) reference base and one alternative allele (guanine (G)) were kept. The loci with more than 4 read count and moderate strand bias (p value ≥ 0.01) were retained. For RNA-seq and DNA-seq, the read count of each site was calculated using samtools and VarScan with minimum base quality 25.

### DNA A-to-G variant filtration

For each individual, high-quality DNA A-to-G variants and the RNA A-to-G loci from Exome-seq and RNA-seq analysis were acquired, respectively, in normal and tumor matched samples separately. To be a true editing event, an A-to-G locus must be specific to the RNA-seq and not in the DNA-seq data. In order to impose this specificity, the loci that were found as DNA A-to-G variants were pruned from the A-to-G loci captured in RNA-seq analysis in the same sample.

### Statistical Filters for Confident RNA Editing Discovery

#### Zero-confidence filter

Many DNA loci corresponding to their RNA A-to-G loci have a low number of alternative G reads simply because the total number of reads is too small. These loci will produce false-positive editing sites. The zero-confidence score measures the statistical confidence in a locus with zero alternative G alleles out of *n* reads. Intuitively our confidence should be lower when *n* is small compared to when *n* is large. More generally, to account for possible sequencing errors, the filter must also allow for small number of alternative G alleles out of *n* reads (i.e. we do not impose exact zeroes). We thus define the zero-confidence score as −10*log_*10*_*(U)*, where *U* is 100(1 − α)% upper-confidence bound of the probability of success based on observing *x* successes out of *n* Bernoulli trials; see Pawitan^34^, Section 5.8. Higher scores mean higher confidence, as typically the case if we observe zero alternative G alleles from larger number of reads. For example, using α = 0.05, if we observe zero alternative G alleles out of n = 5, 10 and 30 reads, the scores are, respectively, equal to 3.46, 5.87 and 10.22. The value α = 0.05 and zero-confidence score cutoff of 10 were applied to retain high-confidence RNA A-to-G editing candidate loci.

#### Odds Ratio and Fisher Exact test on each site

Comparison of the reads statistics from matched tumor and normal samples allows identification of tumor-specific or normal-specific editing events. Odds ratio (OR) and Fisher Exact test p-value between normal and tumor matched samples for a site in an individual were calculated from the 2 by 2 table as described in Fig. 1. Insignificant loci (0.5 < OR < 2.0 or Fisher Exact test p-value > 0.05) were pruned. For strand bias filtration on RNA sequence, Fisher exact test was computed for each loci on a 2 by 2 table made from the number of reads on positive and negative strand for reference and alternative alleles. P value ≥ 0.01 was applied for strand bias on each loci.

#### The use of dbSNP sites to assess false positive rates

Having RNA and DNA sequencing with very high coverage or PCR based confirmation on the same samples is desirable to estimate the reliability of the discovered editing loci. But, in practice, it is impossible to perform these kinds of validation due to unavailability of the identical samples. A priori we do not expect an editing event to occur in previously known SNPs, so we can use SNPs in the dbSNP database (build 151) as negative controls. Moreover, identified A-to-G editing loci that coincide with SNP positions are likely to be DNA variants that have been missed in the variant calling step of the DNA-seq data analysis. In practice we use the dbSNP database as negative controls: a good discovery set should have fewer editing sites within the dbSNP database. In fact dbSNP positions are normally filtered out by some RNA-editing pipelines^12,35,36^, highlighting the common views that they tend to be false positives. However, there are also known editing sites in dbSNP, though they are too few to affect its use as negative controls^37^. So, while we use dbSNP sites to assess false positive rates, for our final candidates we do not rule out dbSNP-sites, as there are possible errors in the dbSNP database^38^.

## Results

### Analysis of TCGA samples

We now describe the results of our analysis of the TCGA breast-cancer matched samples. In total, taken from 60 individuals, 2,967,207 candidate loci (zero-confidence score ≥ 10.0 in both matched samples), only 408,614 loci (13.7%) were still present in dbSNP. An illustration showing the effectiveness of the zero-confidence filter is given by the scatter plots in Fig. 1A.

A total of 20,168 highly confident A-to-G sites (p-value < 0.05 and zero-confidence score ≥ 10.0) were identified from the 60 individuals; 82.5% of these loci were not in dbSNP. The number of A-to-G loci discovered from the whole analysis pipeline is shown in Fig. 1B and C. Figure 1B counts the A-to-G loci captured in at least one individual. Significant A-to-G sites with more editing in tumor (odds ratio ≥ 2.0) or with more editing in normal tissues (odds ratio ≤ 0.5) in any individual are also presented in Fig. 1B. Figure C shows 439 highly confident editing loci discovered in more than 4 individuals out of 60. The number of RNA editing sites after applying CREDO’s filters were presented in Supplementary Table 1. There were 3,866,996 candidate sites were obtained before applying any CREDO’s filter. Each filter contributes in identifying reliable RNA editing events, though Fisher Exact test p-value between normal and tumor samples was the most strict filter. The subsequent analyses will focus on these confident sites that were recurrent in more than 4 individuals.

We next checked if the edited loci are supported by differential expression of ADAR genes which produce the RNA editing enzymes. For each identified site, samples were divided into ‘edited’ and ‘non-edited’ groups. Then, the expression difference of ADAR genes between tumor and normal samples of the ‘edited’ group was measured for each edited loci. In total 210 editing loci out of 439 highly recurrent loci (Fig. 1C) were found to be differentially expressed (DE, Welch’s test P-value < 0.05) in ADAR genes.

### CREDO outperformed three other pipelines in false-positives pruning

False-positive pruning capability is indispensable in RNA-editing discovery because of the genome-wide high-throughput and high-noise nature that makes it susceptible to false positives. In particular, an effective RNA-editing discovery pipeline should be able to discriminate true RNA modifications, which are true positives, from those caused by DNA-level heterozygosity, which are false positives. This problem occurs because heterozygosity calls depend on sufficient reads coverage of the potential editing site in the DNA-sequence data, but inherent non-uniformities in reads coverage make this hard to achieve genome-wide. CREDO was compared with three other methods^12,35,36^ using the defaults filters. In contrast to CREDO, they were designed to identify RNA-editing loci in single samples without matched sample, i.e., normal and tumor samples from identical individual. To make all the methods comparable to each other, CREDO was redesigned to identify RNA editing loci in single sample. For each candidate site, a 2 × 2 table of the number of reference (Adenine) and alternative (Guanine) alleles based on the RNA and DNA sequences from an individual was used to compute odds-ratio (OR) and Fisher Exact test statistics (see the Methods and Materials). The RNA-editing candidates with confidence-zero score on their DNA read counts at least 10.0, and OR among RNA and DNA read counts of 2.0 were judged as confident editing loci in CREDO; see the Methods and Materials section. The other steps before the confidence-zero and OR filters in Fig. 1A are the same as the matched-sample case.

**Figure 2:**
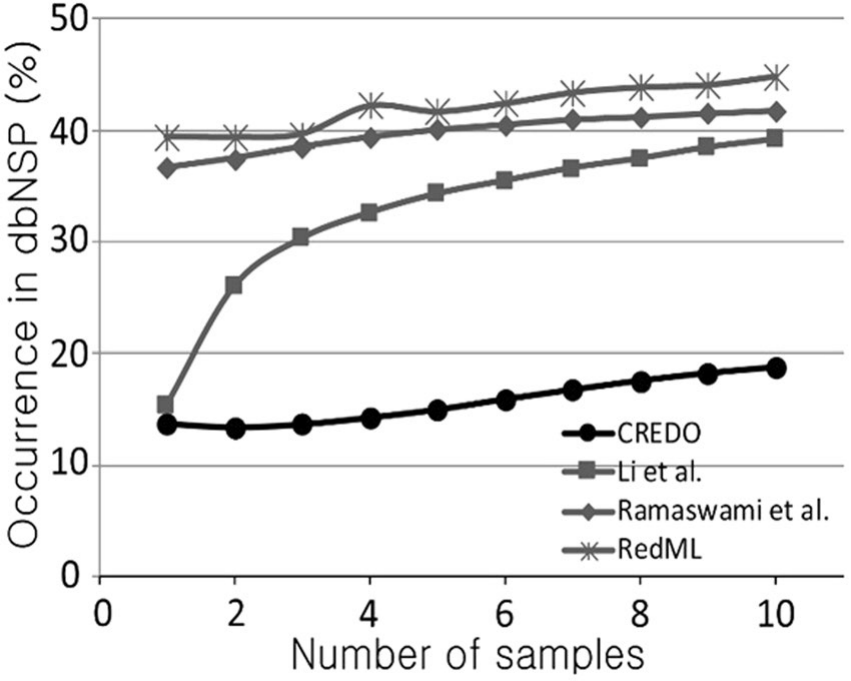
dbSNP overlap percentage of RNA editing candidates discovered by CREDO and the other methods. The x-axis is the number of recurrent samples for the editing sites.

As summarized in Fig. 2, compared to other methods, CREDO showed substantially fewer rates of editing candidates present in dbSNP. The pattern is largely consistent across the number of recurrent sites in multiple individuals. The exact numbers are given in Supplementary Table 2.

### Confident A-to-G Loci residing genes were related with disease

Tables 1–2 list 10 most recurrent potential editing loci out of the confident A-to-G sites in Fig. 1C. Table 1 shows the sites that are also present in dbSNP. (As we previously stated we did not rule out dbSNP-sites, since not all dbSNP-sites are necessarily false positives, as there are possible errors in the dbSNP database^38^.) These identified A-to-G editing loci reside in the genes closely related with immune systems. Table 2 lists highly recurrent potential editing loci from the 60 individuals that are not present in dbSNP. There were 159 confident A-to-G loci (OR ≥2.0 or OR ≤0.5) not in dbSNP. Table 2A and B list the sites with OR ≥2.0 (i.e. higher editing rate in tumors) and OR ≤0.5 (i.e. lower editing rate in tumors), respectively. Intriguingly, Table 2A and 4B are clearly distinct if we compare the corresponding genes and their related diseases. All genes in Table 2A, more edited in tumor samples, are closely related with cancers. For example, DENR and HLA-F were suggested as important players in breast cancer cells^39,40^. NCSTN was found to be a tumor suppressor in NOTCH pathway^41^, and PIK3R2 participates in PI3K/AKT pathway which is most commonly dysregulated in many human cancers^42^. On the other hand, the loci in Table 2B were found in mainly in immune-related genes, while 2 genes are also related with cancers. From these observations, RNA editing events on the loci in Table 2 could be associated with breast cancer prognosis, which we studied next.

**Table 1:** Confident A-to-G loci (Zero-confidence score ≥ 10.0, p-value < 0.05, OR ≥2.0 or OR ≤0.5) identified by CREDO that are also present in dbSNP. These 10 loci are most recurrent among the 60 breast-cancer patients. Sample count is the number of individuals with edited site. Gene is the gene symbol that the site is resided.

Chromosome	Location	Sample Count	Gene	Region	Disease	Gene Description
2	89246858	17	IGKV1-5	Exon		Immunoglobulin Kappa Variable
20	29628273	16	FRG1B	Exon	Prostate cancer, Glioma	FSHD region gene 1 family, member B
2	89567830	16	IGKV1-33	Exon		Immunoglobulin Kappa Variable
2	89246846	15	IGKV1-5	Exon		Immunoglobulin Kappa Variable
2	89442084	14	IGKV3-20	Exon		Immunoglobulin Kappa Variable
2	89384712	14	IGKV3-15	Exon		Immunoglobulin Kappa Variable
20	29623218	13	FRG1B	Exon	Prostate cancer, Glioma	FSHD region gene 1 family, member B
2	89326695	13	IGKV3-11	Exon		Immunoglobulin Kappa Variable
22	23243214	12	IGLC2	Exon	Breast cancer	Immunoglobulin Lambda Constant 2
2	89326707	12	IGKV3-11	Exon		Immunoglobulin Kappa Variable

**Table 2:** Confident A-to-G loci (Zero-confidence score ≥ 10.0, p-value < 0.05) identified by CREDO that are not present in dbSNP. These 10 loci are most recurrent among the 60 breast-cancer patients. (A) OR ≥2.0, meaning higher rate of editing in tumors. (B) OR ≤0.5, meaning lower rate of editing in tumors. Sample count is the number of individuals with edited site. Gene is the gene symbol that the location is resided.

Chromosome	Location	Sample Count	Gene	Region	Disease	Gene Description
**(A)**						
12	125396510	12	UBC	Exon	Apocrine adenoma, Congenital granular cell tumor	Ubiquitin C
12	123253679	11	DENR	3′UTR	Pleomorphic adenoma carcinoma, Breast cancer	Density regulated re-Initiation and release factor
1	160319987	9	NCSTN	Exon	Tumor suppressor	Nicastrin
12	123253657	8	DENR	3′UTR	Pleomorphic adenoma carcinoma, Breast cancer	Density regulated re-Initiation and release factor
17	8280945	7	RPL26	Exon	Diamond-Blackfan Anemia, Conjunctival Cancer	Ribosomal Protein L26
7	74612697	7	GTF2IP1	Exon	Williams-Beuren Syndrome	General Transcription Factor 2I Pseudogene 1
6	29693034	7	HLA-F	Exon	Autoimmune Disease, Breast cancer	Major Histocompatibility Complex
19	18288551	6	PIK3R2	Exon	malignant mixed tumor of corpus uteri, neuronal disease, cancer	Phodphoinositide-3-Kinase Regulatory Subunit 2
22	23040726	6	IGLV2-23	Exon		Immunoglobulin Lambda Variable
22	23101520	6	IGLV2-14	Exon		Immunoglobulin Lambda Variable
**(B)**						
13	46090371	25	COG3	Exon	Breast cancer	Component Of Oligomeric Golgi Complex 3
2	89567797	17	IGKV1-33	Exon		Immunoglobulin Kappa Variable
12	125396510	14	UBC	Exon	Apocrine adenoma, Congenital granular cell tumor	Ubiquitin C
2	89521317	14	IGKV2-28	Exon		Immunoglobulin Kappa Variable
2	89442169	13	IGKV3-20	Exon		Immunoglobulin Kappa Variable
14	106452683	11	IGHV1-2	Exon		Immunoglobulin Heavy Variable
14	106641723	9	IGHV1-18	Exon		Immunoglobulin Heavy Variable
14	106725213	9	IGHV3-23	Exon		Immunoglobulin Heavy Variable
2	89521251	9	IGKV2-28	Exon		Immunoglobulin Kappa Variable
2	89521275	9	IGKV2-28	Exon		Immunoglobulin Kappa Variable

### Survival Analysis

Kaplan-Meier estimate (KM) was used to assess the clinical relevance of RNA editing on disease progression in terms of patient survival. First, for each editing site, an individual is categorized as ‘edited’ if the site is identified as both high-confidence and ADAR-DE; as described above, there are 210 such sites. A total of 34 confident A-to-G editing loci were found to be associated with patient survival (nominal log-rank P-value < 0.05).

**Figure 3:**
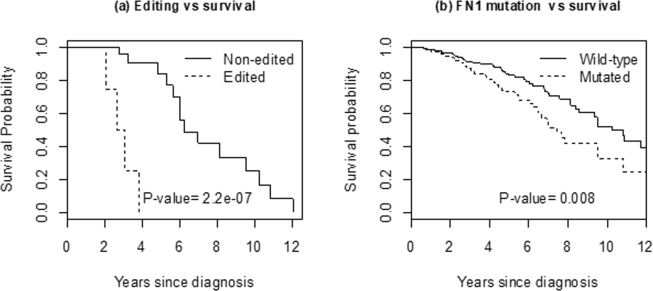
(**a**) Kaplan-Meier curves of breast-cancer patient survival categorized according to 4 top edited loci: Chr2:89544486 (IGKV2-30), Chr2:89185437 (IGKV4-1), Chr2:216236722 (FN1) and Chr19:20727605 (ZNF737). Because they are individually too infrequent, they are combined into a single edited group (n = 16) vs non-edited group (n = 44). (**b**) Corresponding survival curves from FN1-mutated (n = 413) vs wild-type (n = 667) from the full TCGA breast-cancer data.

Figure 3(a) presented the KM survival curves based on the top four edited loci: Chr2:89544486 (IGKV2-30), Chr2:89185437 (IGKV4-1), Chr2:216236722 (FN1) and Chr19:20727605 (ZNF737). Because they are individually too infrequent, these four sites are combined into a single marker, so that an individual is called edited if at least one of the four is edited. The edited group comprises 27% (16/60) of the total patient cohort. Their survival is significantly lower (nominal P-value = 2.2e-7) compared to non-edited group (n = 44). The P-value is highly significant when corrected for multiplicity (207 sites, Bonferroni corrected P-value = 4.6e-5).

Since there is no comparable external data on RNA editing, we instead consider the impact of DNA mutations in these genes among 1080 subjects with breast cancer in the TCGA database. We hypothesize that DNA mutations in these genes would be associated with poor survival. Mutation calls are provided in the TCGA project based on the Mutect algorithm^43^; to increase confidence in the mutation calls, we remove all singleton calls, so each mutation in further analysis is recurrent in at least two individuals. There were no mutations observed in the IGKV.1 and ZNF747 genes, and only two mutations in IGKV2.30. The FN1 gene, however, is frequently mutated, seen in 413 (38%) of the cohort. Figure 3(b) shows that women with FN1-mutation have significantly worse survival (log-rank P-value = 0.008) compared to those with no mutation.

## Discussion

Identification of reliable RNA editing events relevant to disease development is an important and difficult problem. Most of existing approaches is susceptible to high false positives due to numerous technical and biological noises in NGS library preparation, reads mapping, or the mosaic nature of RNA editing itself. We have developed CREDO, an NGS analysis pipeline with novel statistical modules, which integrate DNA- and RNA-seq data from matched normal and tumor tissues from multiple individuals to discover highly confident and reproducible disease-relevant RNA editing loci. The pipeline successfully removed vast majority of false positives, e.g. most of the candidate loci found in the dbSNP database were filtered out without using information from the database itself. CREDO outperformed three other RNA-editing identification pipelines by pruning significantly more false positives.

Basal-like subtype breast-cancer patients with high-level ADAR1 expression had been shown to have more RNA editing and worse clinical outcome^9^. An important role of ADAR1 expression and its function, and the significant association between the RNA editing in genes related to cancer-relevant pathways and clinical outcomes were also previously confirmed^10^. CREDO discovered A-to-G loci that are potentially related with breast cancer development. Some identified RNA-editing loci were found to be differentially expressed in ADAR genes and associated with worse survival. The top 4 editing sites comprise 27% of the subjects, so editing with potential clinical relevance is not a rare event. Because deleterious RNA-editing is biologically similar to deleterious DNA-mutations, we validated the clinical relevance of RNA editing in FN1 gene by showing that subjects with FN1-mutation have significantly worse survival compared to those with no mutation. Among the 1080 TCGA breast-cancer cases, there was little or no DNA mutations in IGKV2.30, IGKV.1 or ZNF747 genes. So, intriguingly for these genes, we might have a deleterious event at purely RNA-level. The FN1 and ZNF737 genes are relatively well-known cancer-related genes. Both were found to be overexpressed in aggressive cancer cells^44–46^. FN1 is known to be overexpressed in aggressive thyroid cancer, and silencing of FN1 expression significantly reduced proliferation, adhesion, migration, and invasion in thyroid cancer cells^44^. FN1 was found overexpressed also in renal cancer cells and reported as a determinant for renal cancer aggressiveness^45^.

CREDO outperformed the other approaches in RNA editing discovery with more editing events and far less false positive rates. In addition, reproducibility and clinical relevance of identified editing events was previously not assessed due to limited focus on a single normal sample. The main strengths of CREDO include the integration of DNA-seq and RNA-seq data from multiple matched tumor-normal pairs, and the use of stringent statistical filters such as zero-confidence and recurrence filters. Without the DNA-seq data from normal tissue, we cannot rule out individual/rare A-to-G SNPs explaining the RNA A-to-G variants. Most of the previous studies have focused on single samples using normal tissues. Recurrence over multiple samples greatly improves the statistical confidence in an editing event and reduces false positive events. The use of tumor tissues allows identification of tumor-specific editing events, which have higher likelihood of being clinically relevant. As the main weakness, our clinical validation results are based on a small sample size. At present, even for a large project such as the TCGA, there are still few samples with complete DNA- and RNA-seq data from the matched tumor-normal pairs. However, this problem is getting continually addressed as the value of having multiple omics from each patient sample is more recognized, so we can expect larger samples in future studies^47^.

## Supplementary information

Supplementary file

## Data Availability

Project name: CREDO. Project home page: https://github.com/biostatUniBS/CREDO.
