# Supplementary material for: CREDO: Highly confident disease-relevant A-to-I RNA-editing discovery in breast cancer
Source: Sci Rep. 2019 Mar 25;9:5064. doi: 10.1038/s41598-019-41294-y (PMC6433923; doi:10.1038/s41598-019-41294-y)
Supplement: Supplementary file 1 — Supplementary file [file 41598_2019_41294_MOESM1_ESM.docx]

**CREDO: Highly confident disease-relevant A-to-I RNA-editing discovery in breast cancer**

Woochang Hwang^1,2†^, Stefano Calza^3,4†^, Marco Silvestri^4,5^, Yudi Pawitan^3*^, Youngjo Lee^1,2^

^1^Data Science for Knowledge Creation Research Center, Seoul National University, Seoul, South Korea.

^2^Department of Statistics, Seoul National University, Seoul, South Korea.

^3^Department of Medical Epidemiology and Biostatistics, Karolinska Insitutet, Stockholm 17177.

^4^Department of Molecular and Translational Medicine, University of Brescia, Italy

^5^Department of Applied Research and Technical Development, Fondazione IRCCS, Istituto Nazionale dei Tumori, Milan, Italy

# Supplementary Information


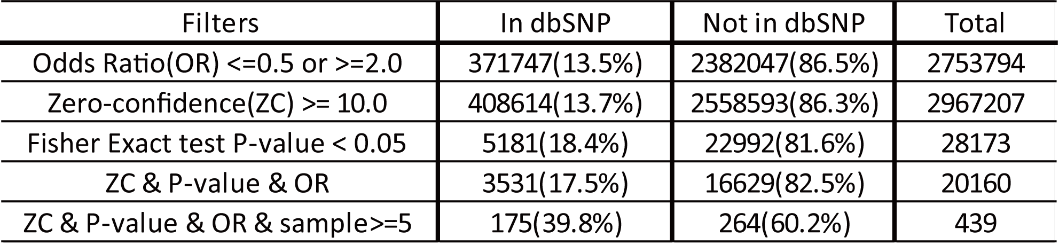


**Supplementary Table 1** Number of RNA editing loci obtained after CREDO’s filters applied. 3,866,996 candidate sites were obtained before applying any CREDO’s filter.


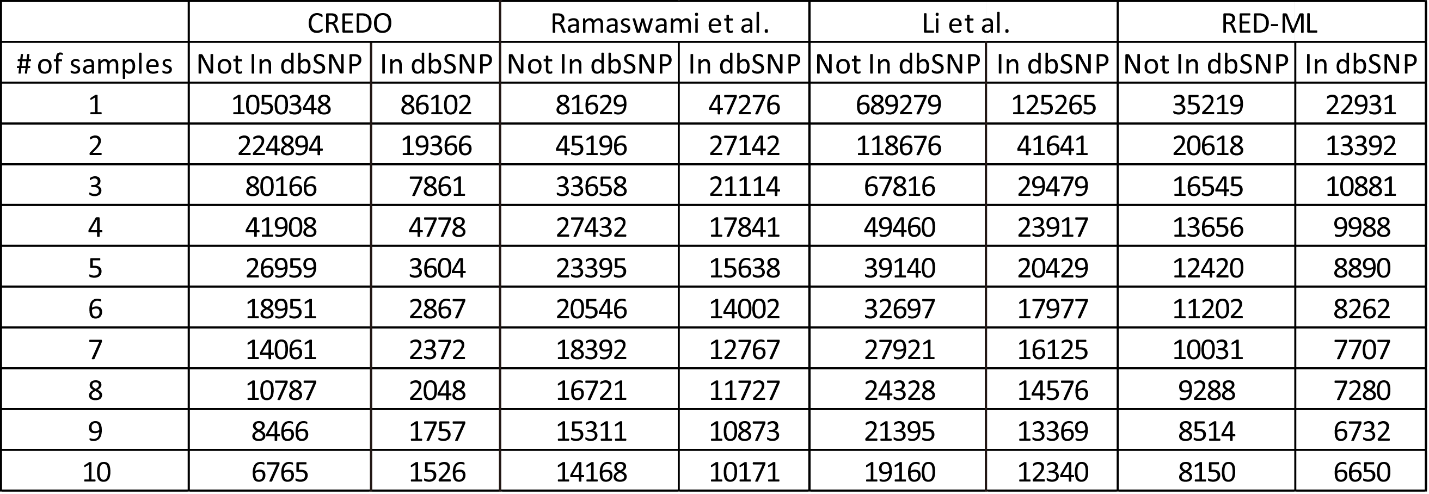


**Supplementary Table 2** Number of RNA editing loci identified by pipelines. First column is the sample count that a site is identified as edited at least certain number of samples.

**References**

Li M, Wang IX, Li Y, Bruzel A, Richards AL, Toung JM, Cheung VG. 2011. Widespread RNA and DNA Sequence Differences in the Human Transcriptome. *Science* **333**: 53-58.

Ramaswami G, Lin W, Piskol R, Tan MH, Davis C, Li JB. 2012. Accurate identification of human Alu and non-Alu RNA editing sites. *Nature Methods* **9**: 579-581.

Xiong H, Liu D, Li Q, Lei M, Xu L, Wu L, Wang Z, Ren S, Li W, Xia M et al. 2017. RED-ML: a novel, effective RNA editing detection method based on machine learning. *Gigascience* **6**: (5):1-8.
